# Temperature- and humidity-modified associations between ambient air pollution and syncope outpatient visits: a time series analysis in Beijing, China

**DOI:** 10.1038/s41598-025-34445-x

**Published:** 2026-01-02

**Authors:** Hong Mu, Yufeng Shi, Jiexin Liu, Tong Guo, Shimeng Liu, Bin Xu, Rongshan Wu, Jian Xu

**Affiliations:** 1 Department of Emergency, Beijing Tiantan Hospital, Capital Medical University, Beijing, 100070 China; 2 The Fifth Medical College, Capital Medical University, Beijing, 100070 China; 3 Neurocardiology Center, Beijing Tiantan Hospital, Capital Medical University, Beijing, 100070 China; 4 Department of Neurology, Beijing Tiantan Hospital, Capital Medical University, Beijing, 100070 China; 5 State Key Laboratory of Environmental Criteria and Risk Assessment, Chinese Research Academy of Environmental Sciences, Beijing, 100012 China; 6 State Environmental Protection Key Laboratory of Ecological Effects and Risk Assessment of Chemicals, Chinese Research Academy of Environmental Sciences, Beijing, 100012 China

**Keywords:** Syncope, Outpatient visit, Urban air pollution, Temperature, Relative humidity, Diseases, Environmental sciences, Health care, Risk factors

## Abstract

Atmospheric factors, particularly air pollution, may act as potential triggers for syncope. In this study, we explored the associations between exposure to air pollution and syncope outpatient visits. Data from syncope outpatients at Beijing Tiantan Hospital between 2014 and 2018, daily average levels of air pollutants, such as particulate matter ≤ 2.5 μm and ≤ 10 μm in diameter (PM_2.5_ and PM_10_), nitrogen dioxide (NO_2_), and sulfur dioxide (SO_2_), along with meteorological factors (temperature and relative humidity (RH)) during the same timeframe, were used to construct the datasets. A time-series study employing a Poisson regression model was conducted to investigate the impact of air pollutants on syncope outpatients. The potential modifying effects of minimum temperature (Temp-Min) and RH were further evaluated by stratifying into four quartiles: low, middle-low, middle-high, and high. The results revealed a significant association between air pollution and syncope risk. Specifically, a 10 µg/m^3^ increase in PM_2.5_, PM_10_, and NO_2_ was associated with significant increases in syncope outpatients, with the risk increasing by 0.41% (95% confidence interval (CI): 0.00%, 0.82%), 0.27% (95% CI: -0.03%, 0.56%), and 1.27% (95% CI: 0.18%, 2.37%), respectively. Subgroup analyses revealed stronger associations in males and older adults (71–80 years) than in females and other age groups. Moreover, lower temperatures (< 9.8 °C) and moderate RHs (25 ~ 50th percentile) aggravated the effects of air pollution on syncope outpatients (*P* < 0.05). This study revealed that the risk of syncope is increased by short-term exposure to air pollution and that this effect is exacerbated by temperature and RH.

**Supplementary Information:**

The online version contains supplementary material available at 10.1038/s41598-025-34445-x.

## Introduction

Syncope is a transient loss of consciousness with global cerebral hypoperfusion accompanied by loss of postural tone, characterized by rapid onset and complete spontaneous recovery^1^. As a common clinical syndrome in everyday medical practice, syncope has an estimated lifetime prevalence of approximately 40% in the general population, accounting for 1%–3% of emergency department visits and 6% of hospital admissions^2^. Given its various clinical presentations and underlying etiologies, syncope is a significant health and social challenge. It not only substantially impairs quality of life but also leads to additional health problems, such as cardiovascular diseases (CVDs)^3^, cerebrovascular diseases (CERs)^4^, cancer^5^, gastrointestinal bleeding^6^, and accidents^7^, particularly among middle-aged and older adults. Although the pathophysiological mechanisms underlying syncope have not been fully clarified, they are related primarily to abnormal regulation of the brain circulatory system^8^.

Global climate change is currently one of the most critical environmental health issues impacting human populations, which is highlighted in the Intergovernmental Panel on Climate Change’s Sixth Assessment Report (IPCC AR6)^9^. The challenges of air pollution and climate change have posed significant threats to global health. Exposure to particulate matter and gaseous pollutants, in conjunction with ambient temperature and relative humidity (RH), may contribute to increased disease incidence^10^, excess mortality^11^, and increased hospital admissions^12^, which increase the global disease burden and stress the importance of health management systems, such as emergency outpatient visits^13,14^.

Air pollution and climate change have become crucial global threats to human health, necessitating investigations to bridge the gap between the environmental and medical fields. Recent explorations have demonstrated that air pollutants and climate change affect the circulatory and neurological systems: CVDs account for 15.9–20.0% of inpatients attributed to PM_2.5_ (particulate matter ≤ 2.5 μm in aerodynamic diameter, PM_2.5_) per year, and air pollution increases the susceptibility to several diseases, including CERs and CVDs, by more than 40% of daily diagnoses^15^. Associations have been reported between nitrogen dioxide (NO_2_) and PM_2.5_ with CVD in outpatients during both short- and long-lag periods^16^. Extreme cold days contributed 9.1 (95% CI, 8.6–9.4) more deaths for every 1000 ischemic strokes^17^.

However, syncope has not been reported in similar studies related to air environmental influences until now. The effects of air pollutants on the circulatory and neurological systems may involve the autonomic nervous system triggering the neural stress response. PM_2.5_ is a prominent risk factor for CVD, possibly affecting the autonomic adrenergic system^18^. The levels of cerebral blood flow and inflammatory factors changed in a PM_2.5_-induced brain damage model^19^. These findings may suggest that syncope is affected by ambient air pollution.

Considering the tremendous disease burden of syncope and its increasing trend in China, we hypothesized that exposure to air pollution and fluctuations in ambient climate change could contribute to syncope incidence. This study aimed to investigate the associations between air pollution, meteorological variables, and syncope outpatients in adults in Beijing from 2014 to 2018. These findings may be important for developing environmental and public health policies to assist vulnerable people in their daily lives and mitigate relevant health effects.

## Materials and methods

### Data for syncope outpatients

The data of daily outpatient visits for syncope from October 1, 2014, to September 30, 2018, were collected from Beijing Tiantan Hospital. The target hospital is a large-scale general hospital specializing in neurological disorders and cerebrovascular diseases in China, with 2.1 million outpatients and emergency visits annually. Patient information included sex, age, residence, dates of hospital visit and diagnosis. The inclusion criteria for patients were as follows: (1) aged between 35 and 80 years, since the percentage of young patients aged 20–35 years was low in our hospital; (2) residing in a radius of 10 km around the hospital in Beijing; (3) the term “syncope” as the primary diagnosis; and (4) not a subsequent visit. Owing to the first occurrence, only the first target hospital visit for syncope was included, which eliminates possible repeated diagnoses for return visits. Syncope diagnoses, identified by the R55 code. X04 within the International Statistical Classification of Diseases and Related Health Problems 10th Revision (ICD-10), were all confirmed by specialists. The data were classified according to age (35–60, 61–70, and 71–80 years) and sex (male or female). All patient information was anonymized prior to data collection. This study was conducted in accordance with the Declaration of Helsinki and was approved by the Ethics Committee of Beijing Tiantan Hospital (Approval No. KY2024-021-02). The need to obtain informed consent was waived by the committee.

### Air quality and meteorological data

Daily meteorological data for the research period were obtained from the Beijing Municipal Environmental Monitoring Center, with 35 monitoring stations distributed in Beijing homogenously. The monitoring station chosen was located at Tiantan Park in Dongcheng District, which is less than 3 km from Beijing Tiantan Hospital. The monitoring station provides ambient air quality data, including hourly concentrations (µg/m^3^) of PM_2.5_, PM_10_, NO_2_, and sulfur dioxide (SO_2_), and daily concentrations of these pollutants were calculated based on 18–24 hours’ valid measurements each day. These daily air pollutant levels were utilized to assess the exposure to air pollution among all syncope patients. Concurrently, daily data on the mean temperature (Temp, ◦C), minimum temperature (Temp-Min, ◦C), mean relative humidity (RH, %), and minimum relative humidity (RH-Min, %) were obtained from the China Meteorological Data Sharing Service System (http://data.cma.cn/).

### Statistical analysis

Among all the residents in Beijing, daily syncope outpatients have a Poisson distribution, so a quasi-Poisson generalized additive model (GAM) was utilized to assess the associations between the concentrations of each air pollutant and the number of syncope outpatients^20,21^. The formula was as follows:

$$\log \left( {E\left( {Y_{t} } \right)} \right) = \alpha + \beta {\text{ }} \cdot Z_{t} + s\left( {time,df} \right) + s\left( {temp,df} \right) + s\left( {RH,df} \right) + DOW$$

where,

- $\:{Y}_{t}$ = daily number of syncope outpatient visits.
- $\:{Z}_{t}$ = daily concentration of the pollutant (e.g., PM_2.5_).
- $\:\beta\:$ = concentration-response coefficient.
- $\:s\left(\right)$ = smooth functions for long-term trend and meteorological variables.
- $\:DOW$ = day-of-week indicator.

The model was adjusted for potential confounders: time trend, RH, day of the week (DOW), temperature, and public holidays. A basic model, excluding air pollutants, was first constructed. The time trend was controlled for via a natural cubic spline function with 8 degrees of freedom (DF) per year to account for seasonality and long-term trends. The temperature and RH were also adjusted via a natural cubic spline function with DF to account for the nonlinear effects of meteorological factors. On the basis of previous studies suggesting delayed associations of air pollution with health outcomes of approximately one week (Yang et al., 2022), we considered both single-day (lag0–lag6) and multiple-day lags (lag01–lag06, representing the average of current and preceding days) for each air pollutant. The means of the current day and six prior days of outpatient visits (lag06) for Temp-Min and RH were also included in the basic model. A natural cubic spline with 2 degrees of freedom was employed as the optimal DF determined by minimizing Akaike’s information criterion^22^. The adjustments of DOW and public holidays aimed to control for potential differences in the number of daily syncope outpatients. Finally, PM_2.5_, PM_10_, NO_2_, and SO_2_ were added to the established model individually to assess the associations of a 10 µg/m^3^ increase in air pollution with syncope outpatients. We further estimated the exposure response relationships between air pollutants and syncope outpatients with smoothing term of 3 DF based on GAM.

We then investigated the possible effect modification stratified by sex (male and female), age (35–60, 61–70, and 71–80 years), and season (“meteorological seasons” and natural seasons). The meteorological seasons were divided into four combinations, namely, the cold/humid season, cold/dry season, warm/humid season, and warm/dry season, on the basis of whether Temp-Min and RH were below or above the median values (median: 9.8 °C for Temp-Min and 51.0% for RH). These four meteorological season categories were then entered as dummy variables into effect modification analyses to estimate their joint influence on the effects of air pollution. The natural seasons were also assessed as potential effect modifiers via a similar approach with dummy variables: spring (March–May), summer (June‒August), fall (September‒November), and winter (December‒February).

We subsequently examined whether the effects of air pollution on syncope outpatients were modified by meteorological factors, including pH and ambient temperature. Temp-Min and RH were classified into four categories by quartiles: low (< 25th percentile), middle-low (25th–50th percentile), middle-high (50th–75th percentile), and high (> 75th percentile), which were added to the model separately as indicator variables. Similar to previous studies, the interaction terms between the indicator variables of Temp-Min or RH and air pollutants were also included in the model, and the effect modifications of Temp-Min and RH were considered statistically significant if the *P* value of the interaction term was less than 0.05^23,24^.

We performed sensitivity analyses to evaluate the stability of the results. Firstly, we explored double-pollutant models, including pairs of pollutants with Spearman correlation coefficients < 0.6, to minimize multi-collinearity. Secondly, we varied the DF in the natural spline functions used for the time trend (7–9), Temp-Min (3–4), and RH (1–3).

Here, the excess risks (ERs) % is calculated directly from the model’s coefficient (β) for each pollutant, using the formula as follows:

ER % = (exp(β × ΔC) – 1) × 100%.

Where ER% is excess risk, β is the model’s coefficient for each pollutant, ΔC is the increment in pollutant concentration. The results are presented as ERs and associated 95% confidence intervals (CIs) per 10 µg/m^3^ increase in PM_2.5_, PM_10_, NO_2_, and SO_2_ at lag01, since lag01 exhibited the maximum impact of air pollution on the days of the syncope outpatients. R software (version 4.3.2) with the “mgcv” and “splines” packages was used for all the statistical analyses. We considered *P* < 0.05 as statistically significant and *P* < 0.1 as potentially significant^25^.

## Results

Table 1 showed the descriptive statistics included daily syncope outpatient data, air pollutant levels, and meteorological variables in Beijing from October 2014 to September 2018. In total, 17,293 outpatients with syncope (35 ≤ age ≤ 80 years), including 6,949 males and 10,344 females, were recorded. On average, 11.84 outpatient adults with syncope as the first diagnosis visited the hospital daily during that period. The patients’ age was 61.06 ± 10.83 years, with no significant difference between the sexes (*P* > 0.05). Females (7.08) and middle-aged adults (35–60 years, 5.79) accounted for the majority of daily average outpatient cases. The number of syncope outpatients roughly changed with season (Figure S1).

**Table 1:** Summary statistics of daily outpatient, air pollutant, and meteorological variables.

Variables	Summary	Mean ± SD	Min	P25	Median	P75	Max
Outcomes	Sum of cases						
Syncope	17,293	11.84 ± 4.01	2	9	11	14	27
Male	6949	4.76 ± 2.33	0	3	5	6	14
Female	10,344	7.08 ± 2.94	0	5	7	9	21
35–60 years	8454	5.79 ± 2.78	0	4	6	7	17
Male	3502	2.4 ± 1.67	0	1	2	3	9
Female	4952	3.39 ± 2.04	0	2	3	5	12
61–70 years	5039	3.45 ± 1.89	0	2	3	4	11
Male	2140	1.46 ± 1.2	0	1	1	2	6
Female	2899	1.98 ± 1.43	0	1	2	3	9
71–80 years	3800	2.6 ± 1.71	0	1	2	4	10
Male	1307	0.89 ± 0.95	0	0	1	1	6
Female	2493	1.71 ± 1.4	0	1	1	3	8
Air pollutants	Number of days						
PM_2.5_ (µg/m^3^)	1398	68.88 ± 63.53	3.26	26.84	50.45	88.02	499.33
PM_10_ (µg/m^3^)	1313	100.76 ± 79.89	1.80	48.91	80.24	126.83	926.11
NO_2_ (µg/m^3^)	1399	48.56 ± 23.51	2.17	32.09	43.27	58.68	170.67
SO_2_ (µg/m^3^)	1399	8.86 ± 9.42	1.29	2.88	5.59	10.79	84.38
Meteorological variables							
Temp (°C)	1461	13.95 ± 11.2	−14.3	2.7	15.6	24.3	32.6
Temp-min (°C)	1461	8.99 ± 11.07	−15.2	−1.7	9.8	19.1	29.1
RH (%)	1461	51.72 ± 20.36	8	35	51	68	99
RH-min (%)	1461	31.36 ± 18.23	5	16	27	45	95

Abbreviations: SD, standard deviation; PM_2.5_ and PM_10_, particulate matter with an aerodynamic diameter less than or equal to 2.5 μm and 10 μm; NO_2_, nitrogen dioxide; SO_2_, sulfur dioxide; Temp, temperature; Temp-Min, minimum temperature; RH, relative humidity; RH-Min, minimum relative humidity.

During the 4-year period, the average pollutant concentrations were as follows: PM_2.5_ (68.88 µg/m^3^), PM_10_ (100.76 µg/m^3^), NO_2_ (48.56 µg/m^3^), and SO_2_ (8.86 µg/m^3^). The average daily temperature was 13.95 °C (minimum: 8.99 °C), and the average daily RH was 51.72% (minimum: 31.36%). In general, the air quality in Beijing is characterized by particularly high levels of PM_2.5_ and PM_10_, with both mean and median concentrations exceeding those of developed countries. Additionally, the NO_2_ concentration was mildly high on most days. In contrast, the SO_2_ concentration was lower than those of the other pollutants, with an even lower median concentration.

Spearman’s correlations were used to explore the relationships between air pollutant levels and meteorological factors (Table S1, Figure S2). Strong positive correlations (*r* > 0.6) were observed between PM_2.5_, PM_10_, and NO_2_, which suggests their potential for co-occurrence. We observed moderate correlations between SO_2_ and PM_2.5_ (*r* = 0.48), PM_10_ (*r* = 0.41), and NO_2_ (*r* = 0.55). Both temperature and Temp-Min exhibited negative correlations with all the measured air pollutants. Both the RH and minimum RH were negatively correlated with only the SO_2_ concentration.

**Fig. 1:**
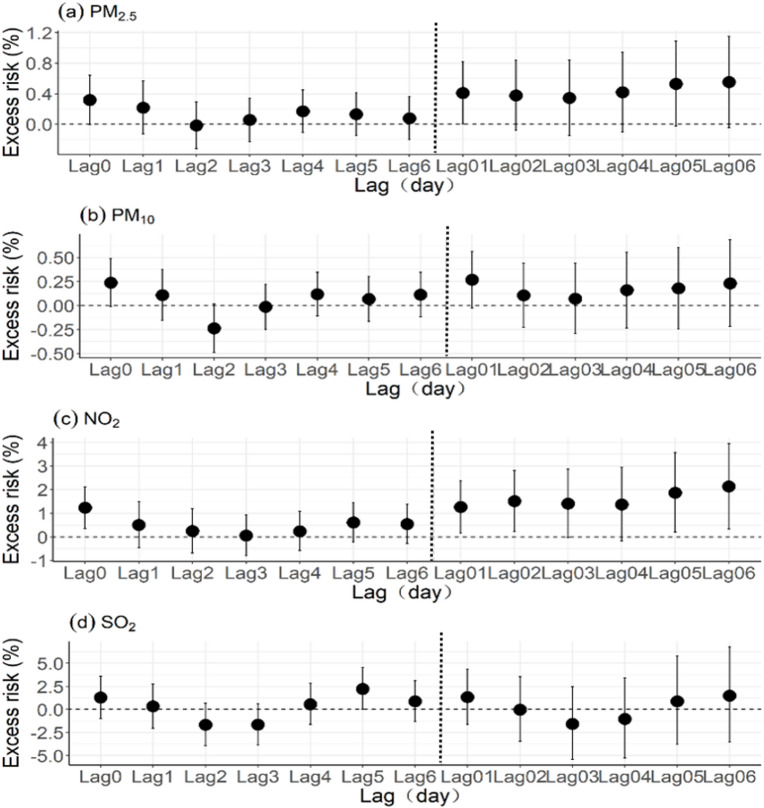
ER% and 95% CI of syncope outpatients associated with each 10 µg/m^3^ increase in each type of air pollution (PM_2.5_, PM_10_, NO_2_ and SO_2)_ on different lag days over the whole research period in Beijing.

Figure 1 shows the exposure‒response relationships between various air pollutants and syncope outpatients over the entire research period. Excess risks of syncope outpatients associated with each 10 µg/m^3^ increase in each type of air pollution (PM_2.5_, PM_10_, NO_2_ and SO_2_) on different lag days over the whole research period in Beijing were shown in Fig. 1. We considered both single-day (Left: lag0–lag6) and multiple-day lags (Right༚lag01–lag06), representing the average of current and preceding days for each air pollutant. Table 2 summarizes the estimated percentage change in the ER associated with each air pollutant at lag01, stratified by sex and age group. Specifically, a 10 µg/m^3^ increase in PM_2.5_ was associated with a 0.41% increase (95% CI: 0.00%, 0.82%) in syncope outpatients. Similar increases were observed for PM_10_ (0.27%, 95% CI: −0.03%, 0.56%) and NO_2_ (1.27%; 95% CI: 0.18%, 2.37%). Subgroup analyses indicated that males and the oldest age group (71–80 years) experienced greater increases in the number of syncope outpatients associated with air pollution than females and the other age groups (35–60 and 61–70 years). For example, among males, a 10 µg/m^3^ increase in PM_2.5_, PM_10_ and NO_2_ corresponded to increases in syncope outpatients of 0.76% (95% CI: 0.15%, 1.38%), 0.51% (95% CI: 0.07%, 0.95%), and 1.76% (95% CI: 0.10%, 3.44%), respectively. For the oldest age group (71–80 years), a 10 µg/m^3^ increase in PM_2.5_, PM_10_ and NO_2_ was associated with an ER of 0.73% (95% CI: −0.08%, 1.55%), 0.70% (95% CI: 0.11%, 1.30%) and 2.10% (95% CI: −0.11%, 4.36%) in syncope outpatients, respectively.

**Table 2:** Excess risk % (95% CI) of outpatients associated with 10 µg/m^3^ increases in PM_2.5_, PM_10_, NO_2_ and SO_2_ (lag01).

Variables		Excess risk,%(95%CI) ^a^							
		PM_2.5_	*P*	PM_10_	*P*	NO_2_	*P*	SO_2_	*P*
Total		0.41 (95%CI: 0.00, 0.82)*	0.048	0.27 (95%CI: −0.03, 0.56)	0.074	1.27 (95%CI: 0.18, 2.37)*	0.023	1.34 (95%CI: −1.61, 4.38)	0.376
Sex	Male	0.76 (95%CI: 0.15, 1.38)*	0.014	0.51 (95%CI: 0.07, 0.95)*	0.023	1.76 (95%CI: 0.10, 3.44)*	0.038	1.58 (95%CI: −2.88, 6.24)	0.494
	Female	0.18 (95%CI: −0.33, 0.69)	0.484	0.11 (95%CI: −0.26, 0.48)	0.565	0.97 (95%CI: −0.38, 2.35)	0.161	1.25 (95%CI: −2.41, 5.05)	0.508
Age	35–60 years	0.31 (95%CI: −0.28, 0.90)	0.303	0.18 (95%CI: −0.25, 0.60)	0.419	0.68 (95%CI: −0.89, 2.27)	0.401	0.52 (95%CI: −3.66, 4.87)	0.811
	61–70 years	0.32 (95%CI: −0.40, 1.04)	0.38	0.09 (95%CI: −0.43, 0.61)	0.734	1.63 (95%CI: −0.28, 3.58)	0.096	2.42 (95%CI: −2.84, 7.96)	0.374
	71–80 years	0.73 (95%CI: −0.08, 1.55)	0.077	0.70 (95%CI: 0.11, 1.30)*	0.02	2.10 (95%CI: −0.11, 4.36)	0.063	2.01 (95%CI: −3.88, 8.26)	0.512

a Results from Poisson regression models, adjusted by time trend, Temp-Min, RH, DOW, and public holidays

* P<0.05

The associations with the ER and its 95% CI per 10 µg/m^3^ increase in PM_2.5_, PM_10_, NO_2_, and SO_2_. We observed that the ER values for syncope outpatients in males were 0.76% (95% CI: 0.15%, 1.38%), 0.51% (95% CI: 0.07%, 0.95%), and 1.76% (95% CI: 0.10%, 3.44%) per 10 µg/m^3^ increase in PM_2.5_, PM_10_ and NO_2_, respectively. The oldest age group (71–80 years) also demonstrated notable associations, with ER values of 0.73% (95% CI: −0.08%, 1.55%), 0.70% (95% CI: 0.11%, 1.30%), and 2.10% (95% CI: −0.11%, 4.36%) for the same increase in the same pollutants.

**Fig. 2:**
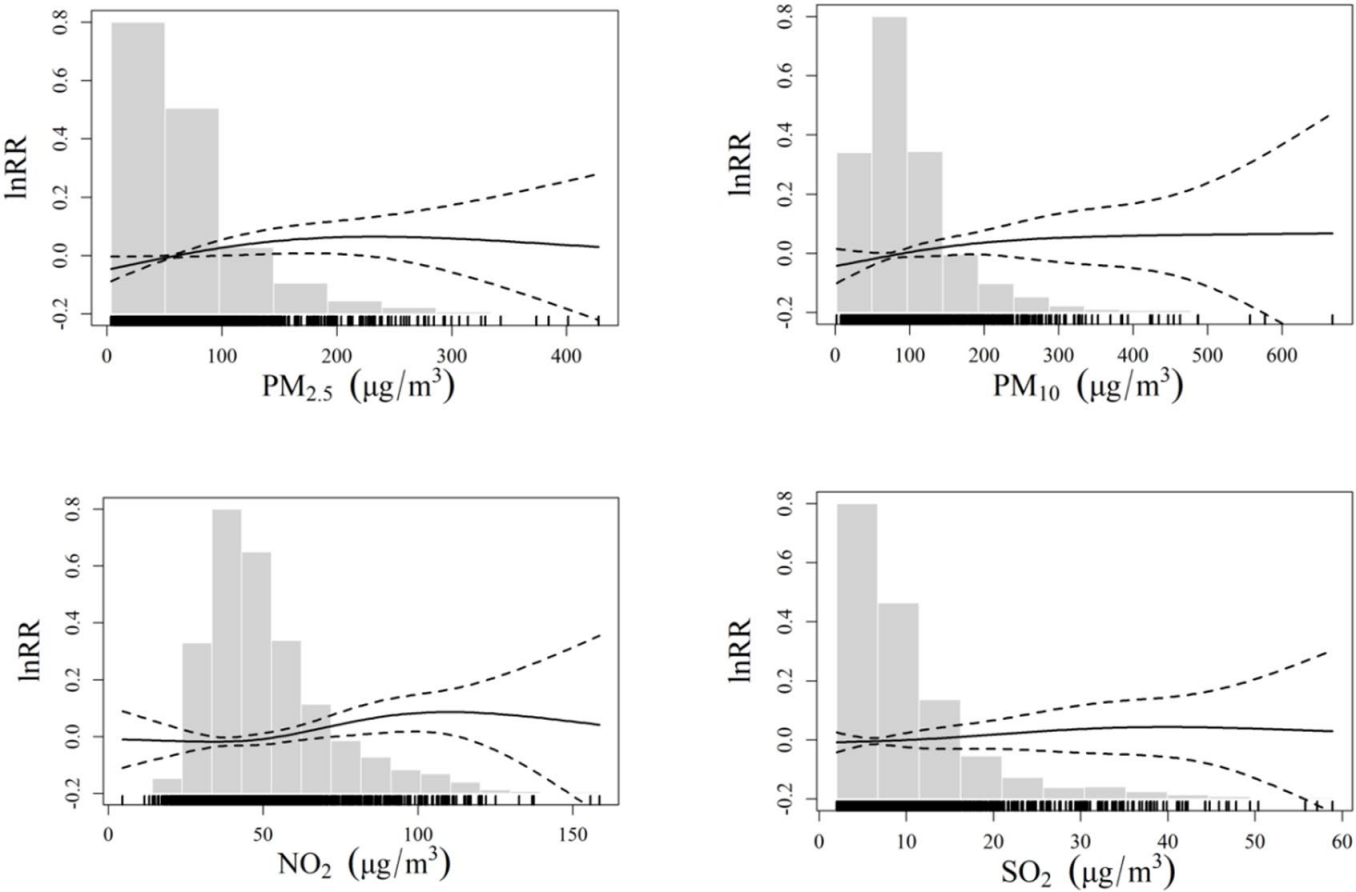
Estimated exposure‒response curves for syncope outpatients associated with each air pollutant (PM_2.5_, PM_10_, NO_2_ and SO_2_) at lag day 1 over the entire research period in Beijing.

Figure 2 shows estimated exposure‒response curves for syncope outpatients associated with each air pollutant (PM_2.5_, PM_10_, NO_2_ and SO_2)_ at lag01 day over the entire research period in Beijing, using the GAM model. The gray background bars represented a frequency histogram of the daily pollutant concentrations during the study period. The exposure-response relationships for PM_2.5_, PM_10_, and NO_2_ displayed an approximately linear trend at lower concentration ranges. However, the curves for PM_2.5_ and PM_10_ exhibited a leveling-off effect at higher concentrations, whereas SO_2_ displayed a leveling-off relationship at all concentrations.

**Fig. 3:**
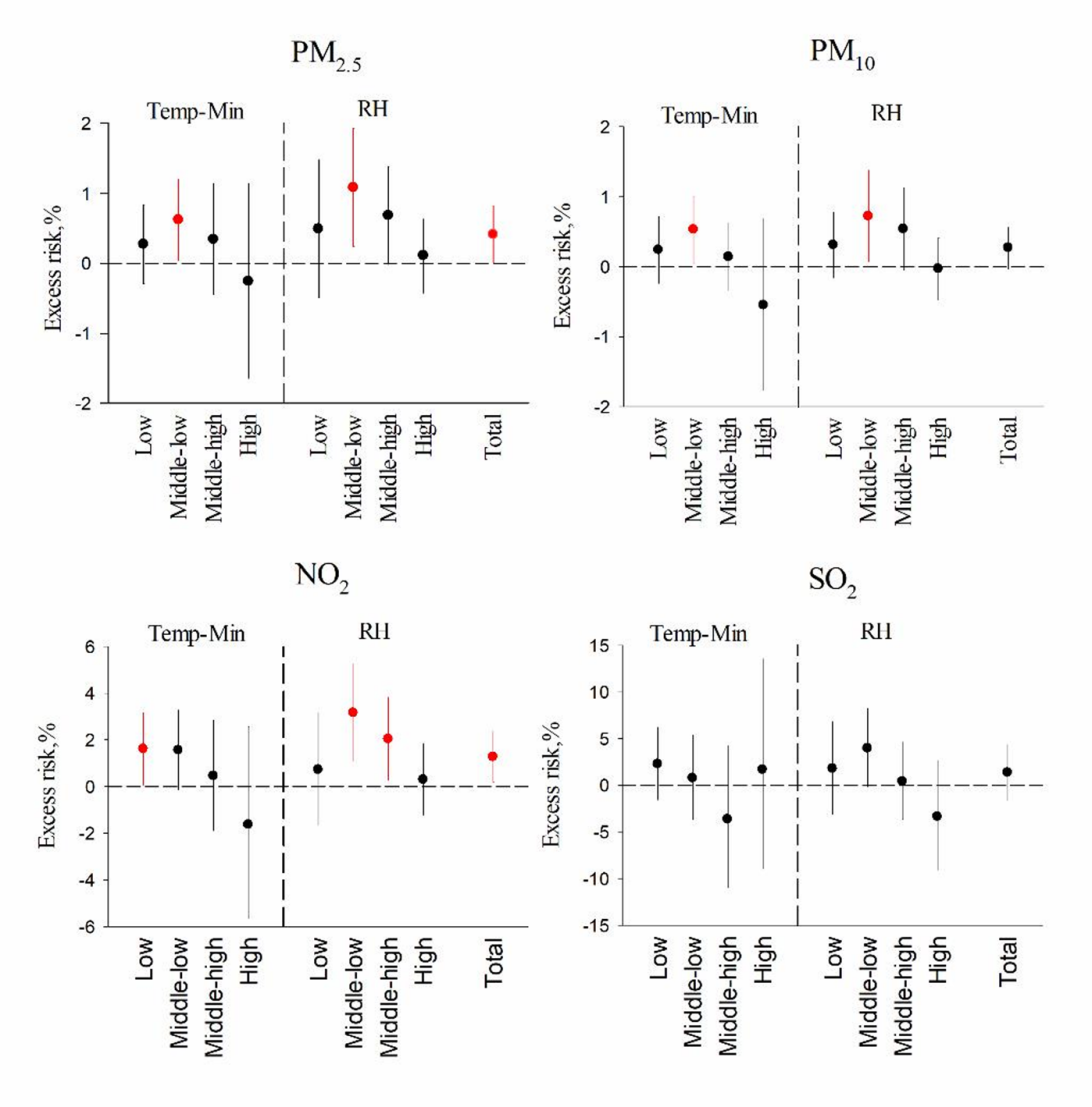
ER percentage (95% CI) of syncope outpatients associated with 10 µg/m^3^ increases in each air pollutant (PM_2.5_, PM_10_, NO_2_, and SO_2_) at lag day 01 stratified by quartiles of Temp-Min or RH. The following quantiles (Q25, Q50, and Q75) were used to define strata: temperature (°C): −1.7, 9.8, and 19.1; relative humidity (%): 35.0, 51.0, and 68.0.

Table 3 shows that lower Temp-Min and higher RH generally corresponded to higher average and median levels of air pollutants, except for SO_2_. Figure 3 and Table S2 illustrate the associations between PM_2.5_, PM_10_, NO_2_ and SO_2_ and syncope outpatients, as modified by Temp-Min and RH. Figure 3 showed that excess risk of outpatients associated with 10 µg/m^3^ increases in PM_2.5_, PM_10_, NO_2_ and SO_2_ (lag01). Generally, stronger associations between PM_2.5_, NO_2_ and syncope outpatients were observed at lower Temp-Min and moderate RH. Quantitatively in Fig. 3; Table 2&S2, a 10 µg/m^3^ increase in PM_2.5_ and PM_10_ in the middle-low Temp-Min quartiles was associated with 0.62% (95% CI: 0.04%, 1.20%) and 0.53% (95% CI: 0.05%, 1.01%) increases in syncope outpatients, respectively. For NO_2_, the increases were 1.60% (95% CI: 0.06%, 3.16%) and 1.55% (95% CI: −0.16%, 3.28%) in the low and middle-low Temp-Min quartiles, respectively. A 10 µg/m^3^ increase in PM_2.5_, PM_10_ and NO_2_ in the middle‒low RH quartiles corresponded to increases of 1.08% (95% CI: 0.24%, 1.93%), 0.72% (95% CI: 0.07%, 1.38%), and 3.15% (95% CI: 1.09%, 5.26%), respectively.

**Table 3:** Median concentrations of PM_2.5_, PM_10_, NO_2_ and SO_2_ in quartiles of Temp-Min and RH.

Effect modifier	Quartile ^a^	Mean (median)(µg/m^3^)			
		PM_2.5_	PM_10_	NO_2_	SO_2_
Temp-min	Low	80.34(46.29)	108.34(73.78)	58.7(51.45)	14.73(10.25)
	Middle-low	77.35(58.5)	113.36(89.92)	53.7(50.88)	9.42(6.14)
	Middle-high	61.04(43.53)	105.51(87.2)	45.7(42.77)	6.39(4.2)
	High	56.33(52.42)	75.58(72.47)	35.78(34.44)	4.76(3.33)
RH	Low	34.01(25.09)	76.12(57.6)	38.92(37.06)	9.11(5.94)
	Middle-low	60.67(51.45)	97.45(86.74)	52.55(48.49)	11.22(7.51)
	Middle-high	78.97(64.81)	107.18(94.61)	50.63(43.45)	9.23(5.65)
	High	102.75(73.25)	128.25(90.63)	52.49(42.67)	5.9(3.47)

a To define strata, we used the following quantiles (Q25.0, Q50.0, and Q75.0): Temp-Min (°C):−1.7, 9.8, and 19.1; RH (%): 35.0, 51.0, and 68.0

Sensitivity analyses revealed that the main effects were robust when the DFs were changed, which means that the associations of different DFs with time trend, Temp-Min, and RH remained relatively stable (Table S3). The sensitivity results for the double-pollutant models using pollutant pairs with Spearman’s correlation coefficients less than 0.6 are presented in Table S4. These analyses indicated that PM_2.5_ and NO_2_ maintained potentially significant associations with syncope outpatients, whereas PM_10_ did not. Specifically, the association between SO_2_ and syncope became non-significant after adjusting for the presence of other pollutants.

Tables S5 and S6 show the effect modifications in the concentrations of the four pollutants on syncope outpatients in different meteorological seasons or the natural season. PM_2.5_, PM_10_, and NO_2_ were significantly associated with syncope outpatients during the cold and dry seasons (Temp-Min < 9.8 °C, RH < 51.0%). The NO_2_ association was notably stronger in winter than in the other seasons.

## Discussion

Syncope presents a veritable challenge to understanding and developing preventive strategies owing to its complicated etiology and multifaceted nature. This study investigated the associations between short-term exposure to air pollution and syncope in outpatients in Beijing from October 2014 to September 2018. Air pollution has been regarded as an urgent global concern recently, with ample evidence linking exposure to elevated levels of air pollution to increased morbidity and mortality in both neurological and circulatory diseases^26^. Syncope has been closely associated with deregulation of the autonomic nervous and circulatory systems^27,28^. To the best of our knowledge, there have been very limited reports investigating syncope in relation to combined exposure to environmental factors such as air pollution, temperature, and humidity until now. Overall, our findings suggest a positive association between air pollution and an increased risk of syncope, which highlights the importance of the significant lag effects of both air pollution and meteorological factors on syncope outpatients. Thus, research on environmental risk factors should receive more attention in the future.

Recently, the incidence of syncope has increased rapidly, affecting approximately 40% of the global population. Epidemiological studies have shown that syncope has a bimodal age distribution, with peaks in adolescence and after 60 years of age^29^. The occurrence of syncope may differ among countries and could be affected by environmental factors in addition to genetic factors^30^. The emergency department of Beijing Tiantan Hospital mainly provides medical services for adults. Syncope in individuals older than 80 years may not follow the same course usually seen in young people, as very old people rarely spend their time outside the house and are unlikely to be affected by environmental air pollution. Young syncope patients aged 20–35 years would not like to visit the outpatient department until they have serious adverse events. Therefore, we focused on middle-aged and older patients (35–80 years) in this research. Generally, syncope is a symptom with a short period of discomfort, and the emergency department and outpatient department in this hospital are both appointment-free during the study period; therefore, outpatient and emergency visits are more suitable and reliable than other departments based on appointment-on services.

Epidemiological investigations have suggested that air pollution is increasingly recognized as a key environmental determinant of neurological and cardiovascular health. NO_2_ and SO_2_ are prevalent gaseous contaminants in the air we breathe. PM_2.5_ and PM_10_ originate from natural and human activities and have received increasing attention during the last 10 years. Our results revealed positive trends in the relationships between air pollution levels and syncope outpatients. We found that 10 µg/m^3^ increases in PM_2.5_, PM_10_, NO_2_, and SO_2_ were associated with 0.41%, 0.27%, 1.27%, and 1.34% increases, respectively, in syncope outpatients. Although the association between air pollution and syncope has not been previously documented, our observations align with existing evidence linking air pollution to other neurological and cardiovascular disorders. For example, Chen et al. demonstrated, in a Nanjing-based time series study, that increases in PM_2.5_ and PM_10_ coincided with increases in daily CVD visits^31^. Similarly, Kim et al. reported that exposure to particulate matter was associated with neural diseases in a Korean population^32^. Costa et al. reported that increased levels of NO_2_ and SO_2_ are significantly correlated with neurodegenerative diseases^33^. Exposure to PM and NO_2_ is linked to stroke-related morbidity and mortality, although the effects may be influenced by specific factors such as the overall air pollution mix (including particle composition) and individual vulnerability (e.g., age or sex)^34^.

Researchers have explored various potential mechanisms to understand the observed link between air pollution and syncope, mainly focusing on inflammation, oxidative stress and immunosuppression^35,36,37^; however, exact explanations have not been well established. Several studies have suggested that the cerebral cortex may be affected by air pollutants, that the neural circuits are deregulated, that the concentration between the synapse and the synaptic cleft is imbalanced, that the vascular smooth muscle becomes desensitized, and that syncope ultimately occurs^38,39^. High NO_2_ concentrations affect vascular smooth muscle cells by inhibiting the activity of nitric oxide synthase, reducing the synthesis of endothelial relaxing factors, and increasing the calcium ion concentration in vascular smooth muscle cells, which results in vascular vasodilation^40,41^. A more comprehensive understanding of the pathophysiological mechanisms linking air pollution to syncope is necessary owing to its complex etiology.

In the gender-specific analyses, the findings revealed significant associations between PM_2.5_, PM_10_, NO_2_ and syncope outpatients among all patients. The associations were stronger in males than in females, which aligns with earlier research on circulatory diseases^42,43^. The observed gender disparity in syncope risk related to air pollution could be attributed to variations in daily routines and occupational exposures. Men spend more outdoor time for their jobs and physical activities, resulting in exposure to more cumulative air pollution^44^. Age-stratified analyses revealed a more significant correlation between air pollution and syncope in older adults (71–80 years) than in middle-aged (35–60 years) and old-aged adults (61–70 years), similar to studies on neural and circulatory diseases^45,46^. Elderly individuals may exhibit increased vulnerability to air pollution-related syncope because of their decreased pulmonary function and aging detoxification systems^47,48^. The nonsignificant associations in the other two groups in the present study are likely because these patients have stronger cardiopulmonary and neural regulatory functions, care more about body dysfunction and visit hospitals with any relevant discomfort^49^. Moreover, the statistical association in older patients (71–80 years) might be explained by the fact that they are unlikely to visit the hospital until dangerous symptoms are unavoidable, as the number of patients in this group is relatively low^50^.

Until now, there has been a scarcity of evidence regarding whether meteorological factors influence the relationship between air pollution and syncope in outpatients. Our results suggest that lower temperatures exacerbate the effects of air pollution on syncope outpatients. A plausible explanation is that lower temperatures increase the harmful effects of PM_2.5_, PM_10_, and NO_2_ on vascular cells, resulting in greater susceptibility to air pollution from the above three sources^51^. Empirical studies exploring how RH affects the impact of air pollution on syncope are limited^52^. The present findings showed that middle-level RH could exacerbate the influence of PM_2.5_, PM_10_, and NO_2_ pollutants on syncope outpatients. Moderate RH is likely to blend PM_2.5_, PM_10_, and NO_2_ pollution in the air, which might indirectly trigger neurological and cardiovascular disorders^53,54^. Furthermore, RH may compromise pulmonary function and influence the elimination of air pollutants (Ji et al., 2020). We noted that higher levels of PM_2.5_, PM_10_, and NO_2_ were predominant at lower temperatures and moderate RHs, while these pollutants were more concentrated during the winter season (Tables S7-S10). This might explain why the greater effects of air pollution on syncope outpatients occurred in the winter, as lower temperatures and moderate RHs made air pollution more serious. A high Temp-Min usually occurs in summer, with the lowest concentration of air pollution, as shown in Tables S7-S9. Nonhigh temperatures can affect the concentrations of air pollutants. During periods with low temperatures, heat is increasingly used, and most air pollutants may accumulate from the combustion of fossil fuels^55^. High RH promotes clouds to produce precipitation in the form of rain or snow, whereas moderate RH enhances the formation of secondary components^56^. These findings might provide some explanations for how meteorological factors influence the effects of air pollution on syncope outpatients. The intrinsic associations may be clarified in future research.

Sensitivity analyses indicated robust associations between PM_2.5_, NO_2_ and syncope risk, even after adjusting for SO_2_. However, the association between SO_2_ and syncope was not significant after adjusting for PM_2.5_, PM_10_ and NO_2_. These findings suggest that PM (PM_2.5_, PM_10_) and NO_2_ play important vital roles in the risk of syncope. Moreover, the association between air pollution and syncope outpatients was significantly greater in the cold/dry season than in the other seasons. In addition, the effect of NO_2_ was significantly greater in winter than in the other seasons, but the difference was not statistically significant in the other seasons. Notably, air pollutant concentrations exhibited a marked seasonal pattern, with higher levels in winter and lower levels in summer (Tables S7-S10). These findings suggest that lower temperatures and moderate RHs may jointly increase the concentrations of air pollutants and increase the adverse health impacts of air pollution on syncope outpatients. Until recently, there has been limited research on how temperature and RH jointly modify the influence of air pollution on syncope. The complicated nature of air pollution in megacities, such as Beijing, makes it challenging to determine the precise contribution of each factor. Thus, further studies should be conducted in a variety of cities with diverse climatic characteristics to determine how air pollution and meteorological factors combine to affect syncope outpatients.

Our present study had several limitations. Firstly, our analysis relied on data from a single tertiary hospital and a fixed-site ambient monitoring station. Although representative for the study area, this may introduce exposure misclassification, as individual mobility patterns, indoor air quality, and precise personal exposure levels were not captured. Secondly, the study focused on adults (35–80 years), excluding adolescents who represent one peak in the bimodal age distribution of syncope. Thirdly, our time-series analysis demonstrated an association between air pollution and syncope outpatient visits, but as an observational and ecological study at the population level, it cannot establish causality. Additionally, individual-level confounders (e.g., socioeconomic status, detailed behavioral patterns) were not available for adjustment. Finally, we did not obtain data on inflammatory and oxidative stress exposure in syncope patients because of a lack of blood test data. Therefore, further research is needed to elucidate the effects of air pollution on syncope more comprehensively.

## Conclusions

This time series study provides novel evidence that short-term exposure to ambient air pollution—specifically, PM_2.5_, PM_10_, and NO_2_—is significantly associated with an increased risk of syncope outpatient visits in Beijing, China. Key findings demonstrate that a 10 µg/m³ increase in PM_2.5_, PM_10_, and NO_2_corresponds to 0.41%, 0.27%, and 1.27% increases in syncope risk, respectively, with heightened susceptibility observed among males and the oldest adults (71–80 years). Notably, lower temperatures (< 50th percentile) and moderate relative humidity amplify the adverse effects of air pollution, underscoring the synergistic role of meteorological factors in exacerbating health outcomes. These findings carry critical implications for public health policy, emphasizing the need for targeted air quality regulations and adaptive strategies to protect vulnerable populations during periods of high pollution and unfavorable weather conditions. The study also highlights the importance of integrating environmental risk assessments into syncope prevention frameworks, particularly in rapidly urbanizing regions with heavy pollution burdens. Future research should prioritize multicenter studies across diverse climatic regions, incorporate personal exposure monitoring, and explore the biological mechanisms linking air pollution to autonomic or cerebrovascular dysfunction.

## Supplementary Information

Below is the link to the electronic supplementary material.

Supplementary Material 1

## Data Availability

The data that support the findings of this study are available upon request from the corresponding author.
