# Supplementary material for: Temperature- and humidity-modified associations between ambient air pollution and syncope outpatient visits: a time series analysis in Beijing, China
Source: Sci Rep. 2026 Jan 2;16:4441. doi: 10.1038/s41598-025-34445-x (PMC12864903; doi:10.1038/s41598-025-34445-x)
Supplement: Supplementary file 1 — Supplementary Material 1 [file 41598_2025_34445_MOESM1_ESM.docx]

**Supplementary materials for**

**Temperature- and Humidity-Modified Associations between Ambient Air Pollution and Syncope Outpatient Visits: A Time series Analysis in Beijing, China**

Hong MU ^1^, Yufeng SHI ^2^, Jiexin LIU ^3^, Tong GUO ^4^, Shimeng LIU ^4^, Bin XU ^1*^, Rongshan WU ^5*^, and Jian XU ^6^^[[1]](#footnote-1)^*

*1 Department of Emergency, Beijing Tiantan Hospital, Capital Medical University, Beijing 100070, China*

*2 The Fifth Medical College, Capital Medical University, Beijing 100070, China*

*3 Neurocardiology Center, Beijing Tiantan Hospital, Capital Medical University, Beijing 100070, China*

*4 Department of Neurology, Beijing Tiantan Hospital, Capital Medical University, Beijing 100070, China*

*5 State Key Laboratory of Environmental Criteria and Risk Assessment, Chinese Research Academy of Environmental Sciences, Beijing 100012, China*

*6 State Environmental Protection Key Laboratory of Ecological Effects and Risk Assessment of Chemicals, Chinese Research Academy of Environmental Sciences, Beijing 100012, China*

Number of pages: 18

Number of Figures: 2

Number of Tables: 10

**Supplementary figures**


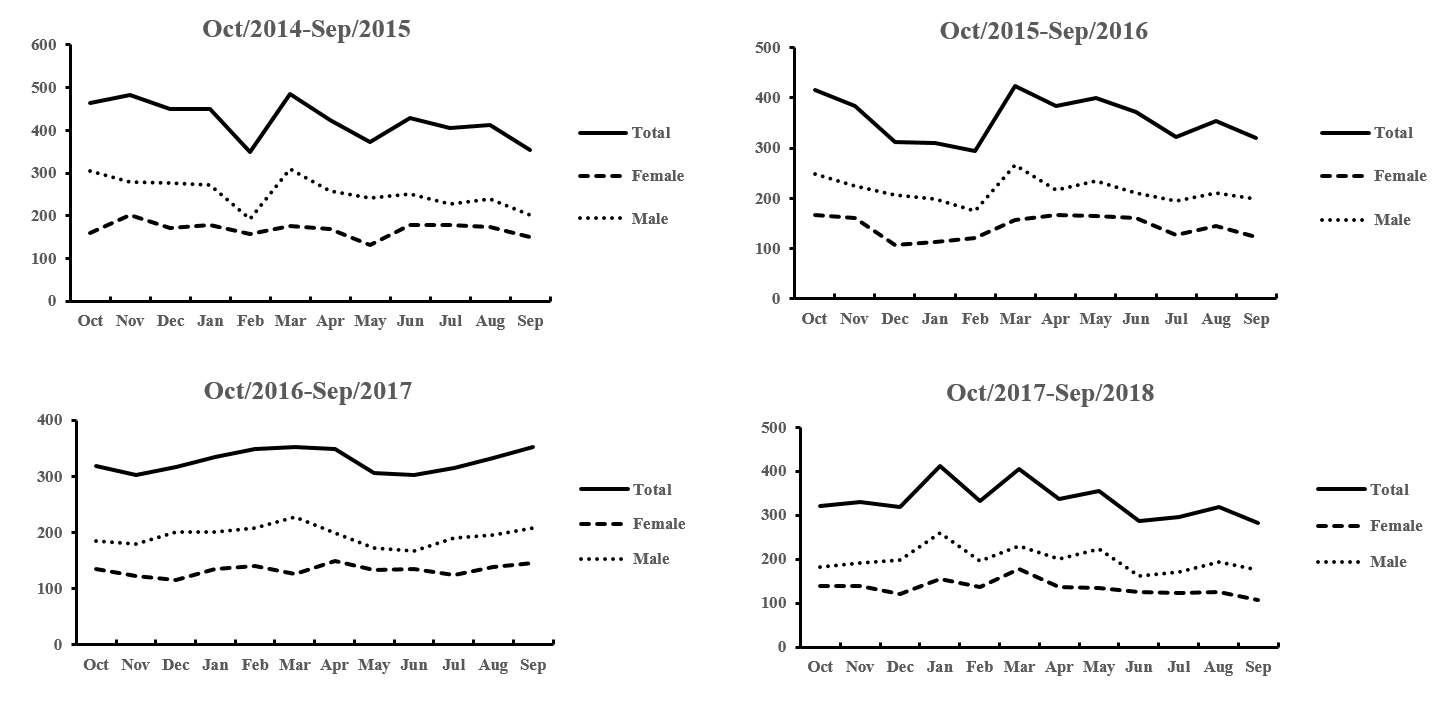


Figure S1. The column chart of syncope outpatients summary from October-2014 to September-2018 by month


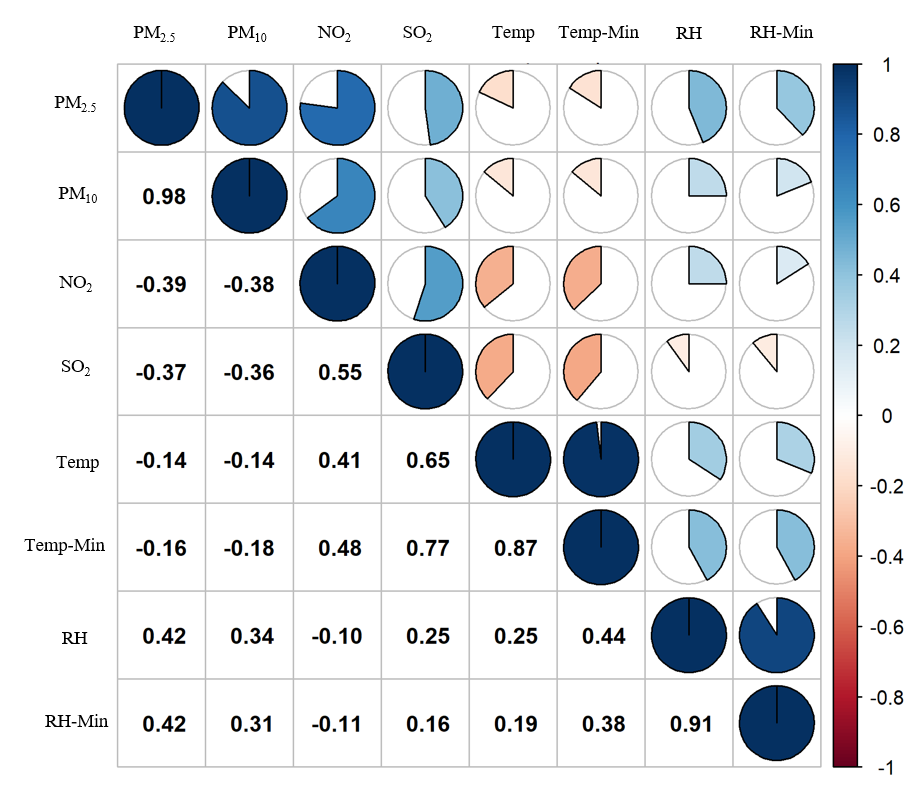


Figure S2. Thermogram of Spearman’s correlation coefficient matrix of air pollutants and meteorological variables in Beijing from October 2014 to September 2018

**Supplementary tables**

Table S1 Spearman’s correlation between daily air pollutants, temperature and relative humidity.

| Variables | PM_2.5_ | PM_10_ | NO_2_ | SO_2_ | Temp | Temp-Min | RH | RH-Min |
| --- | --- | --- | --- | --- | --- | --- | --- | --- |
| PM_2.5_ | 1** | 0.87** | 0.77** | 0.48** | -0.18** | -0.16** | 0.44** | 0.38** |
| PM_10_ | 0.87** | 1** | 0.65** | 0.41** | -0.14** | -0.14** | 0.25** | 0.19** |
| NO_2_ | 0.77** | 0.65** | 1** | 0.55** | -0.36** | -0.37** | 0.25** | 0.16** |
| SO_2_ | 0.48** | 0.41** | 0.55** | 1** | -0.38** | -0.39** | -0.1** | -0.11** |
| Temp | -0.18** | -0.14** | -0.36** | -0.38** | 1** | 0.98** | 0.34** | 0.31** |
| Temp-Min | -0.16** | -0.14** | -0.37** | -0.39** | 0.98** | 1** | 0.42** | 0.42** |
| RH | 0.44** | 0.25** | 0.25** | -0.1** | 0.34** | 0.42** | 1** | 0.91** |
| RH-Min | 0.38** | 0.19** | 0.16** | -0.11** | 0.31** | 0.42** | 0.91** | 1** |

***P* < 0.01 *0.01≤ *P* <0.05

Table S2 Excess risk % (95% CI) of syncope outpatients for associated with 10 μg/m^3^ increases in PM_2.5_, PM_10_, NO_2_ and SO_2_ (lag01) modified by quartiles of Temp-Min and RH in Beijing, 2014-2018.

| Effect modifier | | Excess risk,% (95%CI) ^a^ | | | | | | | |
| --- | --- | --- | --- | --- | --- | --- | --- | --- | --- |
|  |  | PM_2.5_ | *P* | PM_10_ | *P* | NO_2_ | *P* | SO_2_ | *P* |
| Temp- Min | Low | 0.27 (95%CI: -0.29, 0.84) | 0.348 | 0.24 (95%CI: -0.24, 0.72) | 0.328 | 1.60 (95%CI: 0.06, 3.16)* | 0.042 | 2.26 (95%CI: -1.54, 6.20) | 0.248 |
|  | Middle- low | 0.62 (95%CI: 0.04, 1.20)* | 0.036 | 0.53 (95%CI: 0.05, 1.01)* | 0.031 | 1.55 (95%CI: -0.16, 3.28) | 0.076 | 0.74 (95%CI: -3.69, 5.36) | 0.749 |
|  | Middle- high | 0.34 (95%CI: -0.44, 1.14) | 0.393 | 0.14 (95%CI: -0.34, 0.62) | 0.576 | 0.45 (95%CI: -1.89, 2.84) | 0.711 | -3.66 (95%CI:-10.97, 4.26) | 0.355 |
|  | High | -0.26 (95%CI: -1.64, 1.13) | 0.710 | -0.55 (95%CI: -1.77, 0.68) | 0.376 | -1.64 (95%CI: -5.67, 2.57) | 0.441 | 1.66 (95%CI: -8.97,13.52) | 0.771 |
| RH | Low | 0.49 (95%CI: -0.49, 1.48) | 0.326 | 0.31 (95%CI: -0.16, 0.78) | 0.197 | 0.72 (95%CI: -1.66, 3.15) | 0.557 | 1.76 (95%CI: -3.06, 6.81) | 0.481 |
|  | Middle- low | 1.08 (95%CI: 0.24, 1.93)* | 0.012 | 0.72 (95%CI: 0.07, 1.38)* | 0.031 | 3.15 (95%CI: 1.09, 5.26)** | 0.003 | 3.94 (95%CI: -0.16, 8.21) | 0.06 |
|  | Middle-high | 0.68 (95%CI: -0.01, 1.38) | 0.054 | 0.54 (95%CI: -0.04, 1.13) | 0.067 | 2.03 (95%CI: 0.27, 3.83)* | 0.024 | 0.40 (95%CI: -3.64, 4.62) | 0.848 |
|  | High | 0.11 (95%CI: -0.41, 0.63) | 0.683 | -0.03 (95%CI: -0.48, 0.41) | 0.89 | 0.29 (95%CI: -1.21, 1.82) | 0.708 | -3.38 (95%CI: -9.07, 2.67) | 0.267 |

^a^ To define strata, we used the following quantiles (Q25.0, Q50.0, Q75.0): Temp-Min (°C): -1.7, 9.8, 19.1; RH (%): 35.0, 51.0, 68.0.

Table S3 Sensitivity analyses of the associations between 10μg/m^3^ increases in PM_2.5_, PM_10_, NO_2,_ SO_2_ (lag01) and syncope outpatients for by different degree of freedom for calendar time, Temp-Min and RH in Beijing, China, 2014–2018.

| Variables | Excess risk,% (95%CI) | | | |
| --- | --- | --- | --- | --- |
|  | PM_2.5_ | PM_10_ | NO_2_ | SO_2_ |
| Main | 0.41 (95%CI: 0.00,0.82) | 0.27 (95%CI:-0.03,0.56) | 1.27 (95%CI: 0.18,2.37) | 1.34 (95%CI:-1.61,4.38) |
| Calendar time |  |  |  |  |
| 7 | 0.38 (95%CI:-0.02,0.79) | 0.27 (95%CI:-0.03,0.56) | 1.13 (95%CI: 0.06,2.20) | 1.45 (95%CI:-1.46,4.45) |
| 9 | 0.36 (95%CI:-0.05,0.78) | 0.24 (95%CI:-0.06,0.54) | 1.17 (95%CI: 0.07,2.29) | 1.21 (95%CI:-1.75,4.25) |
| Temp-Min |  |  |  |  |
| 3 | 0.40 (95%CI: 0.00,0.81) | 0.26 (95%CI:-0.04,0.55) | 1.23 (95%CI: 0.13,2.33) | 1.21 (95%CI:-1.75,4.26) |
| 4 | 0.39 (95%CI:-0.02,0.80) | 0.25 (95%CI:-0.04,0.54) | 1.19 (95%CI: 0.09,2.29) | 1.11 (95%CI:-1.84,4.16) |
| Temp |  |  |  |  |
| 3 | 0.36 (95%CI:-0.07,0.79) | 0.23 (95%CI:-0.08,0.54) | 0.95 (95%CI:-0.19,2.11) | 0.71 (95%CI:-2.23,3.73) |
| 4 | 0.34 (95%CI:-0.09,0.77) | 0.22 (95%CI:-0.09,0.53) | 0.87 (95%CI:-0.28,2.03) | 0.58 (95%CI:-2.36,3.61) |
| RH-Min |  |  |  |  |
| 3 | 0.43 (95%CI: 0.04,0.81) | 0.29 (95%CI: 0.00,0.57) | 1.35 (95%CI: 0.31,2.39) | 1.84 (95%CI:-1.05,4.83) |
| 1 | 0.46 (95%CI: 0.08,0.84) | 0.31 (95%CI: 0.02,0.59) | 1.45 (95%CI: 0.45,2.46) | 2.28 (95%CI:-0.50,5.14) |
| RH |  |  |  |  |
| 3 | 0.41 (95%CI: 0.00,0.82) | 0.27 (95%CI:-0.03,0.56) | 1.27 (95%CI: 0.17,2.38) | 1.33 (95%CI:-1.65,4.41) |
| 1 | 0.45 (95%CI: 0.05,0.86) | 0.29 (95%CI:-0.01,0.58) | 1.48 (95%CI: 0.43,2.54) | 2.20 (95%CI:-0.59,5.08) |

Table S4 Excess risk % (95% CI) of syncope outpatients associated with 10 μg/m^3^ increases in PM_2.5_, PM_10_ and SO_2_ (lag01) in Beijing, China, 2014–2018. Results from two-pollutant models.

| Pollutant | Model^a^ | ER(95%CI) | *P* |
| --- | --- | --- | --- |
| PM_2.5_ | Main | 0.41 (95%CI: 0.00,0.82)* | 0.048 |
|  | Adjusted SO_2_ | 0.42 (95%CI:-0.04,0.89) | 0.077 |
| PM_10_ | Main | 0.27 (95%CI:-0.03,0.56) | 0.074 |
|  | Adjusted SO_2_ | 0.26 (95%CI:-0.06,0.58) | 0.107 |
| NO_2_ | Main | 1.27 (95%CI: 0.18,2.37)* | 0.023 |
|  | Adjusted SO_2_ | 1.38 (95%CI: 0.11,2.68)* | 0.034 |
| SO_2_ | Main | 1.34 (95%CI:-1.61,4.38) | 0.376 |
|  | Adjusted PM_2.5_ | -0.13 (95%CI:-3.44,3.29) | 0.94 |
|  | Adjusted PM_10_ | 0.20 (95%CI:-2.95,3.46) | 0.902 |
|  | Adjusted NO_2_ | -0.60 (95%CI:-3.98,2.90) | 0.732 |

^a^ two pollutant models were limited to pollutants with Spearman correlation coefficients <0.6.

Table S5 Excess risk % (95% CI) of outpatients associated with 10 μg/m^3^ increases in PM_2.5_, PM_10_, NO_2_ and SO_2_ (lag01) modified by climate season^a^ in Beijing, China, 2014–2018.

|  | Excess risk,% (95%CI) ^a^ | | | | | | | | |
| --- | --- | --- | --- | --- | --- | --- | --- | --- | --- |
|  | Cold and dry season | *P* | Cold and humid season | *P* | Warm and dry season | *P* | Warm and humid season | *P* |  |
| PM_2.5_ | 0.97 (95%CI: 0.06, 1.89)* | 0.038 | 0.51 (95%CI:-0.14, 1.16) | 0.127 | 0.50 (95%CI:-0.85, 1.86) | 0.472 | 0.16 (95%CI:-0.61, 0.94) | 0.685 |  |
| PM_10_ | 0.88 (95%CI: 0.21, 1.56)* | 0.011 | 0.27 (95%CI:-0.30, 0.84) | 0.355 | 0.31 (95%CI:-0.26, 0.88) | 0.294 | 0.02 (95%CI:-0.63, 0.66) | 0.961 |  |
| NO_2_ | 2.19 (95%CI: 0.17, 4.25)* | 0.034 | 1.25 (95%CI:-0.78, 3.31) | 0.232 | -1.14 (95%CI:-5.12, 3.00) | 0.584 | 0.47 (95%CI:-2.08, 3.09) | 0.72 |  |
| SO_2_ | 2.76 (95%CI:-1.62, 7.34) | 0.221 | 1.75 (95%CI:-3.64, 7.44) | 0.532 | 0.00 (95%CI:-9.28,10.22) | 0.999 | 1.10 (95%CI:-7.92,11.00) | 0.819 |  |

a To define strata, we divided into four groups by median of Temp-Min (℃): 9.8 and RH (%): 51.0 (cold and dry season: Temp-Min<9.8℃ RH<51.0%; cold and humid season: Temp-Min<9.8℃ RH≥51.0%; warm and dry season: Temp-Min≥9.8℃ RH<51.0%; warm and humid season: Temp-Min≥9.8℃ RH≥51.0%).

Table S6 Excess risk % (95% CI) of outpatients associated with 10 μg/m^3^ increases in PM_2.5_, PM_10_, NO_2_ and SO_2_ (lag01) modified by season^a^ in Beijing, China, 2014–2018.

| Pollutant | Excess risk,% (95%CI) | | | | | | | | |
| --- | --- | --- | --- | --- | --- | --- | --- | --- | --- |
|  | Spring | *P* | Summer | *P* | Fall | *P* | Winter | *P* |  |
| PM_2.5_ | 0.47 (95%CI: -0.44, 1.40) | 0.314 | 0.02 (95%CI: -1.55, 1.61) | 0.983 | 0.46 (95%CI: -0.18, 1.11) | 0.161 | 0.33 (95%CI: -0.56, 1.23) | 0.465 |  |
| PM_10_ | 0.26 (95%CI: -0.24, 0.75) | 0.308 | 0.00 (95%CI: -1.39, 1.41) | 0.999 | 0.43 (95%CI: -0.11, 0.98) | 0.12 | 0.16 (95%CI: -0.57, 0.90) | 0.67 |  |
| NO_2_ | 1.50 (95%CI: -1.18, 4.25) | 0.275 | -0.61 (95%CI: -5.27, 4.29) | 0.804 | 0.10 (95%CI: -1.73, 1.97) | 0.915 | 2.19 (95%CI: 0.05, 4.37) * | 0.045 |  |
| SO_2_ | -0.12 (95%CI: -6.06, 6.20) | 0.97 | 3.15 (95%CI: -9.81,17.97) | 0.651 | -4.64 (95%CI:-11.56, 2.83) | 0.218 | 0.20 (95%CI: -4.11, 4.72) | 0.928 |  |

^a^ Season was divided into Spring(March-May), Summer(June-August), Fall(September-November), Winter(December-February).

Table S7 Month me(di)an concentrations and frequency of PM_2.5_, stratified by quartiles^a^ of Temp-Min and RH in Beijing, China, 2014–2018.

| Season | Month | PM_2.5_ (μg/m^3^) | | | | | | | | |
| --- | --- | --- | --- | --- | --- | --- | --- | --- | --- | --- |
|  |  | Mean (Median) | Mean (Frequency) | | | | | | | |
|  |  |  | Temp-Min | | | | RH | | | |
|  |  |  | Low | Middle Low | Middle High | High | Low | Middle Low | Middle High | High |
| Spring | March | 86.2(73.6) | 61.6(20) | 91.2(87) | 92.6(8) | -- | 43.6(56) | 103.4(35) | 178.1(19) | 92.8(5) |
|  | April | 64.2(58.7) | -- | 58.1(55) | 69.9(60) | -- | 35.9(38) | 71.9(39) | 89.3(34) | 45.5(4) |
|  | May | 55(45.1) | -- | 31.8(7) | 54.2(85) | 68.7(17) | 45.5(37) | 54.6(44) | 69.2(22) | 64(6) |
| Summer | June | 54.3(47.7) | -- | -- | 37.4(45) | 65.4(69) | 31.4(18) | 43.5(36) | 64.6(42) | 75(18) |
|  | July | 58.5(52.2) | -- | -- | 27.8(4) | 59.6(110) | 8.3(1) | 40.6(9) | 63.4(42) | 58.6(62) |
|  | August | 42.1(34.2) | -- | -- | 18.2(11) | 44.4(111) | 8.3(1) | 17.5(15) | 39.9(48) | 50.8(58) |
| Fall | September | 47.4(38.8) | -- | 28.7(4) | 42.4(77) | 59.6(38) | 9.9(9) | 17.3(24) | 47.9(46) | 73.4(40) |
|  | October | 79.3(54.3) | -- | 51.2(67) | 112.9(56) | -- | 11.6(12) | 38.1(14) | 56.2(34) | 113.9(63) |
|  | November | 89.2(65.9) | 82.2(36) | 92.3(84) | -- | -- | 17.8(21) | 57.1(27) | 91.7(27) | 140.4(45) |
| Winter | December | 101.8(61.2) | 98.6(101) | 119(19) | -- | -- | 27.2(42) | 61.4(33) | 142.7(20) | 247.9(25) |
|  | January | 79.3(45.1) | 79.4(115) | 77.6(6) | -- | -- | 33.2(58) | 78.7(37) | 131(14) | 243.9(12) |
|  | February | 66.9(40.2) | 63(82) | 80.3(24) | -- | -- | 37.5(65) | 82.3(25) | 176.7(10) | 138.6(6) |
| Total | Total | 68.9(50.5) | 80.3(354) | 77.3(353) | 61(346) | 56.3(345) | 34(358) | 60.7(338) | 79(358) | 102.7(344) |

^a^ To define strata, we used the following quantiles (Q25.0, Q50.0, Q75.0): Temp-Min (°C): -1.7, 9.8, 19.1; RH (%): 35.0, 51.0, 68.0.

Table S8 Month me(di)an concentrations and frequency of PM_10_, stratified by quartiles^a^ of Temp-Min and RH in Beijing, China, 2014–2018.

| Season | Month | PM_10_ (μg/m^3^) | | | | | | | | |
| --- | --- | --- | --- | --- | --- | --- | --- | --- | --- | --- |
|  |  | Mean (Median) | Mean (Frequency) | | | | | | | |
|  |  |  | Temp-Min | | | | RH | | | |
|  |  |  | Low | Middle Low | Middle High | High | Low | Middle Low | Middle High | High |
| Spring | March | 132.8(109) | 79.8(20) | 131(82) | 304(7) | -- | 107.2(56) | 136.1(35) | 205.7(18) | -- |
|  | April | 116.2(109.4) | -- | 107.8(53) | 123.7(59) | -- | 92(38) | 137.1(39) | 121.5(34) | 38.1(1) |
|  | May | 115.1(99.8) | -- | 90.8(6) | 116.3(82) | 117.5(17) | 125.2(37) | 112.4(43) | 111(21) | 72(4) |
| Summer | June | 80.5(79.3) | -- | -- | 60.2(40) | 93.6(62) | 76.1(18) | 78.4(36) | 84.7(39) | 79.5(9) |
|  | July | 70.9(66.9) | -- | -- | 49.3(4) | 71.8(102) | 23.2(1) | 65.2(9) | 83.3(41) | 63.5(55) |
|  | August | 59.2(49.1) | -- | -- | 41.8(10) | 60.8(108) | 34.3(1) | 38.9(15) | 64.2(47) | 60.9(55) |
| Fall | September | 70.3(55.1) | -- | 58.6(4) | 66.2(73) | 79.7(37) | 40.4(9) | 42.2(24) | 76.4(45) | 88.9(36) |
|  | October | 111.8(83.5) | -- | 80.7(62) | 156.5(43) | -- | 36.6(11) | 65.9(13) | 88.1(33) | 157.7(48) |
|  | November | 123(94.3) | 118.9(33) | 124.8(75) | -- | -- | 47.8(21) | 95.4(27) | 124.1(26) | 190.5(34) |
| Winter | December | 134.2(84.2) | 133(100) | 141.5(16) | -- | -- | 58.8(42) | 88.8(32) | 188(19) | 290.7(23) |
|  | January | 104.2(70.5) | 104.5(113) | 94.2(4) | -- | -- | 56.6(57) | 102.7(37) | 165.5(13) | 300.7(10) |
|  | February | 91.4(60.4) | 85(78) | 113.1(23) | -- | -- | 62.7(65) | 105.2(24) | 209.1(8) | 239.5(4) |
| Total | Total | 100.8(80.2) | 108.3(344) | 113.4(325) | 105.5(318) | 75.6(326) | 76.1(356) | 97.5(334) | 107.2(344) | 128.2(279) |

^a^ To define strata, we used the following quantiles (Q25.0, Q50.0, Q75.0): Temp-Min (°C): -1.7, 9.8, 19.1; RH (%): 35.0, 51.0, 68.0.

Table S9 Month me(di)an concentrations and frequency of NO_2_, stratified by quartiles^a^ of Temp-Min and RH in Beijing, China, 2014–2018.

| Season | Month | NO_2_ (μg/m^3^) | | | | | | | | |
| --- | --- | --- | --- | --- | --- | --- | --- | --- | --- | --- |
|  |  | Mean (Median) | Mean (Frequency) | | | | | | | |
|  |  |  | Temp-Min | | | | RH | | | |
|  |  |  | Low | Mid Low | Mid High | High | Low | Mid Low | Mid High | High |
| Spring | March | 55.6(54.1) | 48.5(20) | 57.3(87) | 55.1(8) | -- | 43.2(56) | 64(35) | 76.6(19) | 56.9(5) |
|  | April | 45.4(43.9) | -- | 45.4(55) | 45.5(60) | -- | 38(38) | 51.3(39) | 48.1(34) | 36.4(4) |
|  | May | 39.2(37.5) | -- | 40.6(7) | 39.3(85) | 37.9(17) | 34.9(37) | 43.3(44) | 37.9(22) | 40(6) |
| Summer | June | 37.2(35.6) | -- | -- | 35.7(45) | 38.1(69) | 41.7(18) | 39(36) | 36.1(42) | 31.3(18) |
|  | July | 33.5(33.2) | -- | -- | 38.7(4) | 33.3(111) | 41.1(1) | 41(9) | 33.7(42) | 32.1(63) |
|  | August | 35(34.3) | -- | -- | 38.8(11) | 34.7(111) | 41.8(1) | 39(15) | 36.4(48) | 32.8(58) |
| Fall | September | 44.8(43.1) | -- | 44.9(4) | 46.5(77) | 41.2(38) | 32.6(9) | 39.6(24) | 49(46) | 45.7(40) |
|  | October | 54.9(50.2) | -- | 48(67) | 63(56) | -- | 32.8(12) | 48.5(14) | 55.9(34) | 59.9(63) |
|  | November | 59.8(54.7) | 59.9(36) | 59.7(84) | -- | -- | 33.2(21) | 58.6(27) | 66.5(27) | 68.9(45) |
| Winter | December | 67.9(64) | 68.3(101) | 65.8(19) | -- | -- | 39.1(42) | 63(33) | 91.8(20) | 103.6(25) |
|  | January | 60.8(54.6) | 60.8(115) | 61.4(6) | -- | -- | 42.6(58) | 68(37) | 75.6(14) | 109.7(12) |
|  | February | 46.4(41.1) | 45.9(82) | 48.1(24) | -- | -- | 37.7(65) | 58(25) | 67.6(10) | 56.7(6) |
| Total | Total | 48.6(43.3) | 58.7(354) | 53.7(353) | 45.7(346) | 35.8(346) | 38.9(358) | 52.6(338) | 50.6(358) | 52.5(345) |

^a^ To define strata, we used the following quantiles (Q25.0, Q50.0, Q75.0): Temp-Min (°C): -1.7, 9.8, 19.1; RH (%): 35.0, 51.0, 68.0.

Table S10 Me(di)an concentrations of PM_2.5_, PM_10_, NO_2_ and SO_2_ stratified by climate season and season in Beijing, China, 2014–2018.

| Group ^a^ | Mean (Median) (μg/m^3^) | | | |
| --- | --- | --- | --- | --- |
|  | PM_2.5_ | PM_10_ | NO_2_ | SO_2_ |
| Cold and dry season | 54.21 (41.45) | 86.18 (70.51) | 48.35 (46.38) | 11.55 (8.3) |
| Cold and humid season | 120.2 (103.34) | 152.58 (131.48) | 68.78 (66.11) | 12.65 (8.94) |
| Warm and dry season | 48.04 (40.6) | 100.31 (85.33) | 41.4 (40.4) | 6.95 (5.23) |
| Warm and humid season | 64.75 (58.1) | 87.15 (77.41) | 41.06 (37.77) | 5.11 (3.67) |
|  |  |  |  |  |
| Spring | 68.37 (58.16) | 120.17 (107.6) | 46.78 (44.76) | 9.88 (8.06) |
| Summer | 51.38 (46.94) | 69.88 (68.48) | 35.23 (34.27) | 4.51 (3.38) |
| Fall | 71.79 (53.58) | 100.14 (80.65) | 53.03 (49.62) | 6.11 (4.45) |
| Winter | 83.75 (56.62) | 112.75 (81.39) | 58.89 (52.46) | 14.98 (11.1) |

^a^ To define strata, we divided into four groups by median of Temp-Min (℃): 9.8 and RH (%): 51.0 (cold and dry season: Temp-Min<9.8℃ RH<51.0%; cold and humid season: Temp-Min<9.8℃ RH≥51.0%; warm and dry season: Temp-Min≥9.8℃ RH<51.0%; warm and humid season: Temp-Min≥9.8℃ RH≥51.0%). Season was divided into Spring(March-May), Summer(June-August), Fall(September-November), Winter(December-February).

1. * Corresponding authors. Bin Xu, Email: [ttyy_xu@163.com](mailto:ttyy_xu@163.com), Rongshan WU, Email: [wu.rongshan@craes.org.cn](mailto:wu.rongshan@craes.org.cn), Jian XU, [xujian@craes.org.cn](mailto:xujian@craes.org.cn). [↑](#footnote-ref-1)
